# Validation of a Home Food Environment Instrument Assessing Household Food Patterning and Quality

**DOI:** 10.3390/nu13113930

**Published:** 2021-11-03

**Authors:** Katherine J. Barrett, Sarah K. Hibbs-Shipp, Savannah Hobbs, Richard E. Boles, Susan L. Johnson, Laura L. Bellows

**Affiliations:** 1 Department of Pediatrics, Section of Nutrition, University of Colorado Anschutz Medical Campus, Aurora, CO 80045, USA; katherine.barrett@cuanschutz.edu (K.J.B.); richard.boles@cuanschutz.edu (R.E.B.); susan.johnson@cuanschutz.edu (S.L.J.); 2 Department of Food Science and Human Nutrition, Colorado State University, Fort Collins, CO 80523, USA; sarah.hibbs-shipp@colostate.edu (S.K.H.-S.); savannah.hobbs@colostate.edu (S.H.); 3 Division of Nutritional Sciences, Cornell University, Ithaca, NY 14853, USA

**Keywords:** home food environment, home food inventory, home food availability, food environment quality, FoodAPS

## Abstract

The home food environment (HFE) is associated with dietary intake; yet measuring HFE quality often requires burdensome collection of detailed inventories. This project evaluated the capacity of the Home Inventory to Describe Eating and Activity, version 2 (Home-IDEA2) to capture HFE quality by measuring the presence or absence of household foods. Validity was tested using a modified application of the Healthy Eating Index-2010 (HEI). Comparative data were drawn from the National Food Acquisition and Purchase Survey (FoodAPS) Food-at-Home Public Use File. HEI scores were calculated for 4202 households in FoodAPS using Home-IDEA2 inventories and full reported inventories. Paired t-tests compared: (1) estimated vs. total edible grams (EEG; TEG); (2) limited vs. all reported foods; and (3) EEG + limited foods vs. TEG + all reported foods. Sensitivity and range of scores were compared. Mean HEI scores for Home-IDEA2 were higher (*p* < 0.003) than FoodAPS: (1) 51.6 ± 16.1 vs. 49.6 ± 18.1 (food amounts); (2) 53.5 ± 15.8 vs. 49.8 ± 15.4 (food items); (3) 55.5 ± 15.7 vs. 49.8 ± 15.4 (full instrument); differences were small. Scores demonstrated comparable sensitivity and range. The study found that the Home-IDEA2 can capture HFE quality adequately with low data collection burden.

## 1. Introduction

The home food environment (HFE) is receiving increasing attention as an important factor in the development of food preferences and eating habits and as a contributor to obesogenic environments. The socio-ecological model (SEM) describes how health outcomes can be shaped by various levels of influence, from the individual to governmental policy [1]. In the context of SEM, the HFE is a modifiable target for nutritional interventions, especially those aiming to prevent childhood obesity [2,3,4]. The HFE, and specifically home food availability, has consistently been associated with dietary intake and quality among both adults and children. For example, household availability of food groups such as fruits, vegetables, and dairy has been positively associated with consumption of those food groups, whereas the presence of sugar-sweetened beverages has been associated with lower diet quality [4,5,6,7,8].

Home food availability and the associated household food patterning, defined by quantity and types of foods present, are important aspects of the HFE [9,10]. There are a variety of ways to measure household food patterning, including via self-reported and observer-completed questionnaires, scanned barcodes, and weighed foods [11,12]. One such assessment is the Home Inventory to Describe Eating and Activity, version 2 (Home-IDEA2), a tool that was developed in collaboration with target audiences and has been tested for validity (content, internal criterion, and construct) and reliability (between independent raters and participants) in assessing food, activity, and electronic home environments among families with limited resources [11,13,14]. The Home-IDEA2 includes a brief inventory of individual food items (e.g., carrots or whole wheat bread) and categories of food items (e.g., citrus fruits) that are commonly found in U.S. households. The inventory can be completed by participants or researchers by simply recording the presence or absence of the listed food items. The Home-IDEA2 has been used by researchers and participants in Colorado who have a wide range of education, household resources, and live in rural and urban settings; and it has been translated into Spanish and tested among Spanish-speaking participants (specifically of Mexican heritage). Because the Home-IDEA2 does not require sophisticated measurement instruments such as barcode scanners and scales, it is a tool that can capture household food patterning with relatively low participant and researcher burden. The capacity of the Home-IDEA2 to capture the quality of household food patterning has been assessed only in small samples and requires additional testing in nationally-representative samples to determine its broader applicability.

The quality of food patterning can be assessed using tools such as the Healthy Eating Index (HEI), a standard density-based pattern measure by which foods are scored according to how they align with the Dietary Guidelines for Americans (DGA) [15,16,17]. The HEI contains component scores that compare food and nutrient intake to recommendations in the DGA as well as adequacy, moderation, and total scores that measure overall diet quality (Table 1), where higher scores indicate higher diet quality. Component scores are classified under the concepts of Adequacy and Moderation, where Adequacy represents foods that contribute to higher scores through higher intake, and Moderation represents foods that contribute to higher scores through lower intake [16]. The HEI-2010 algorithm has previously been applied to the Home-IDEA2 to assign an overall quality score for the home food environment [14]. While this method has been psychometrically tested and internal validity has been established, external criterion validity using a nationally representative dataset has not yet been established.

The National Food Acquisition and Purchase Survey (FoodAPS) is a nationally representative survey of foods found in 4826 diverse households in the United States (U.S.). The survey was conducted by the USDA Economic Research Service (ERS) and Food and Nutrition Service (FNS) between April 2012 and January 2013 [18]. FoodAPS includes households of all income levels: those participating in the Supplemental Nutrition Assistance Program (SNAP), those with incomes at or below 185% of the poverty guideline but not participating in SNAP, and those with incomes equal to or greater than 185% of the poverty guideline. FoodAPS captured the types and quantity of food as well as the frequency of purchasing and acquisition of food items among U.S. households. Data were collected by documenting scanned barcodes, product descriptions, and household locations. Data collection required participation by all household members for all eating and food acquisition occasions over a period of 7 days. FoodAPS datasets have been used to assign household food quality scores by applying the HEI-2010 algorithm [19]. Other researchers have used these HEI scores in order to understand differences between households that had children with and without obesity and family food decision making [20,21]. Because of these previous applications of HEI scores to participating FoodAPS households and the status of FoodAPS as the gold standard for household food availability, the FoodAPS Food-at-Home Public Use File dataset was used to test whether the low-burden Home-IDEA2 could yield HFE information comparable to the collection-intensive FoodAPS methods.

**Figure 1:**
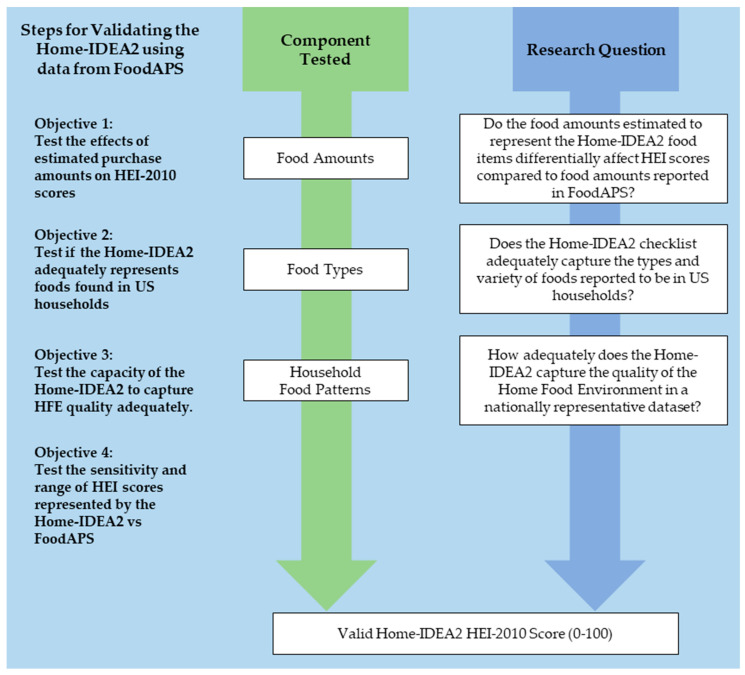
Summary of testing external validity for HEI scores for Home-IDEA2 against HEI scores for FoodAPS, and the validation questions guiding each objective. HEI: Healthy Eating Index. Home-IDEA2: Home Inventory for Describing Eating and Activity, version 2. FoodAPS: National Food Acquisition and Purchase Survey.

The goal of this study was to test the capacity of the Home-IDEA2 to capture the quality of household food patterning by examining the external criterion validity of assigning an HEI-2010 score to the Home-IDEA2. External criterion validity was tested by comparing the HEI component and total scores calculated for the Home-IDEA2 inventory to the FoodAPS full inventory. The present study includes the following objectives: (1) validating selection of amounts of representative foods, (2) validating the capacity of the Home-IDEA2, which includes both individual and categories of food items found in the HFE, to adequately capture overall food patterning in the HFE, (3) validating performance of the Home-IDEA2 in a large, nationally-representative dataset, and (4) examining the range and sensitivity of the component and total scores to detect differences in the HFE (Figure 1).

## 2. Materials and Methods

### 2.1. Calculation of the Home-IDEA2 HEI Scores

The Home-IDEA2 includes 104 items representing various foods commonly found in U.S. households [13]. Each item response is binary (present in the home, not present in the home). Some items indicate the presence of a single food (e.g., carrots or whole wheat bread) and some items indicate the presence of a range of foods (e.g., citrus fruits and frozen sweet treats). Comparative data for the Home-IDEA2 were drawn from the FoodAPS Food-at-Home Public Use File. Prior to use, the Food-at-Home file was evaluated for missingness. Households were removed from the dataset if they did not report any foods or if reported foods did not include total edible grams (TEG). The final analytic sample included 4202 households, each of which contained at least 1 food code and corresponding food amount.

To compare the Home-IDEA2 limited inventory with the FoodAPS full inventory for Objectives 2 and 3, food codes reported in the FoodAPS dataset were mapped to the appropriate corresponding Home-IDEA2 item. For example, food codes that represented citrus fruits (oranges, tangerines, mandarins, lemons, limes, grapefruit, etc.; all forms raw, frozen, canned, with or without syrup) were mapped to the Home-IDEA2 item “citrus fruits” and to the corresponding representative food item (as described in Hibbs-Shipp et al.) [14]. Overall, 1630 of the 3112 unique food codes in the FoodAPS dataset mapped to the 104 Home-IDEA2 items. The Home-IDEA2 did not measure food quantities in the household (only presence or absence). Thus, the realistic purchase amount (i.e., the typical amount purchased) for each Home-IDEA2 item was estimated by triangulating the FoodAPS mean, median, and mode of reported total edible gram (TEG) weights for each item. Typical consumer package sizes were estimated based on internet searches and converted to estimated edible grams (EEG) to be compared against reported TEG. For example, the realistic purchase amount of milk (all varieties) was one gallon or 3.8 L. A complete description of the identification of representative foods and food amounts can be found in Hibbs-Shipp et al. [14].

The HEI-2010 algorithm was selected for application because it was released when FoodAPS data were collected [18,19], and choosing this strategy was consistent with prior research estimating HEI scores utilizing FoodAPS data [20,21]. The HEI-2010 algorithm was applied in several unique ways to establish scores that could be used to test external criterion validity. The method of assignment is defined in each objective below. Each objective compares HEI scores for the same households.

### 2.2. Objective 1: Representation of Food Amounts

Because the Home-IDEA2 measures only the presence or absence of foods in the household, overestimation of EEG for any given food could potentially inflate the density-based HEI scores. Therefore, the first objective was to test if an HEI score based on EEG calculated for the Home-IDEA2 yielded results similar to an HEI score based on amounts reported by FoodAPS households. To do this, only households that included at least one of the 104 food codes selected to represent the Home-IDEA2 items were included in the analysis (N = 4074) (see Hibbs-Shipp et al. for detailed explanation of the selection process) [14]. First, the HEI algorithm was applied to each participating household using the calculated EEG (HEI_EEG_) [14]. Next, the HEI algorithm was applied to the same participating households using TEG from the FoodAPS dataset (HEI_TEG_).

### 2.3. Objective 2: Representation of Food Types

The second objective was to test whether the limited number of food items captured by the Home-IDEA2 adequately represented the patterning of foods reported through a complete home inventory. Each household (N = 4202) was assigned an HEI score based on the total edible gram amounts (HEI_TEG_) in two ways: first the 1630 food codes represented by the Home-IDEA2 were mapped to the corresponding representative food items for the HEI calculation, and second all 3112 food codes in the FoodAPS dataset, including the additional 1482 food codes that did not map to a Home-IDEA2 item, were included in the HEI calculation [14].

### 2.4. Objective 3: Home Food Environment Quality

To test how the Home-IDEA2 limited inventory performed against a full household inventory in a nationally representative dataset, Home-IDEA2 inventories were created for each of the 4202 households included from the FoodAPS dataset. Each of the 1630 food codes included in Objective 2 was mapped to one of the 104 representative food items for the Home-IDEA2. HEI scores were applied to the Home-IDEA2 inventories using EEG, resulting in an HEI_EEG_ score for each household. Additionally, each household was assigned an HEI_TEG_ score based on the full food inventory and TEG (as calculated in Objective 2). The HEI_EEG_ scores for the Home-IDEA2 limited inventory were compared to the HEI_TEG_ scores for the FoodAPS full inventory.

### 2.5. Objective 4: Sensitivity and Range

To test sensitivity and range, the scores calculated in Objective 3 were split into nine percentiles that approximated the ranges expected within a normal distribution (1st, 5th, 10th, 25th, 50th, 75th, 90th, 95th, 99th). Mean scores were compared at each percentile (sensitivity) and across percentile distribution (range).

### 2.6. Statistical Analyses

Datasets were built using SAS (version 9.4; SAS Institute, Inc., Cary, NC, USA), and statistical analyses were completed using SPSS (version 24, IBM Corp., Armonk, NY, USA). Differences between characteristics of households included the analytic sample (N = 4202) and the FoodAPS full sample (N = 4826) were compared using Chi-square and Mann-Whitney U-tests. For each set of HEI comparisons (food amounts, food types, and household food patterning), Pearson’s correlations tested the relationships between component and total scores. Paired t-tests were used to compare the difference between means. The percent difference between means for each component and total score was calculated by dividing the mean difference by the maximum possible value for the respective component or total score. To understand the practical meaning of differences between the Home-IDEA2 and FoodAPS mean HEI scores, dietary intake equivalents for each HEI component score were calculated based on the effect of a 1-point change on dietary intake equivalents, using the HEI-2010 standard for maximum score as the referent (Table 1) [16].

## 3. Results

### 3.1. Sample Description and General Overview

Households included in the present sample had more people than households from the full FoodAPS sample and had larger family sizes (Table 2). The present sample included higher proportions of White and Asian/Native Hawaiian/Pacific Islander primary respondents, and a lower proportion of Black primary respondents. A higher proportion of primary respondents had bachelor’s degrees vs. high school degrees or equivalent. A higher proportion of households reported high adult food security (vs. very low), food sufficiency (enough but not always wanted foods vs. sometimes not enough), and were from the Western (vs. Southern) region of the U.S. There were no significant differences in the proportion of Hispanic respondents, employment, target household group, or participation in the Supplemental Nutrition Assistance Program for Women, Infants, and Children (WIC).

Mean HEI total scores were significantly higher (*p* < 0.003) when calculated using the subset of food items and EEG representing the Home-IDEA2 limited inventory vs. the full set of reported food items and TEG reported in the FoodAPS dataset: 51.6 ± 16.1 vs. 49.6 ± 18.1 respectively for food amounts comparisons (Objective 1); 53.5 ± 15.8 vs. 49.8 ± 15.4 respectively for food items comparisons (Objective 2); 55.5 ± 15.7 vs. 49.8 ± 15.4 respectively for HFE quality comparisons (Objective 3).

### 3.2. Objective 1: Representation of Food Amounts

When comparing the HEI_EEG_ based on EEG for the Home-IDEA2 104 representative food items and HEI_TEG_ that measured amounts reported in FoodAPS, correlations were high (Table 3). Correlations ranged from 0.64 (Sodium) to 0.93 (Whole Grains). Differences between mean component and total scores were significant at *p* ≤ 0.003, with the exception of Sodium (*p* = 0.01) and Fatty Acid Ratio (*p* = 0.77) (Table 4). These differences were small yet significant due to the power to detect very small differences in a sample size this large. The percent difference between the means ranged from −6% difference (Refined Grains) to 7% difference (Total Vegetables, Dairy) (Table 4). The highest practical differences in dietary intake equivalents were 0.09 cup/21 mL (Dairy) and 0.15 oz/4.25 g (Refined Grains).

### 3.3. Objective 2: Representation of Food Types

Correlations between scores for the Home-IDEA2 reduced inventory pattern (1630 food codes that mapped to the 104 food items) and the FoodAPS full household inventory pattern (both measured by HEI_TEG_) were high (Table 3). Correlations ranged from 0.62 (Sodium) to 0.96 (Total Fruit). Differences between mean component and total scores were significant at *p* ≤ 0.003. The percent difference between the means ranged from −5% (Greens and Beans) to 11% (Sodium) (Table 4). The highest practical differences in dietary equivalents were 0.07 cup/17 mL (Dairy) and 0.13 oz/3.68 g (Refined Grains).

### 3.4. Objective 3: Household Food Patterning

Correlations between component and total scores between the Home-IDEA2 limited inventory (HEI_EEG_, 1630 food codes mapped to 104 food items) and the FoodAPS full inventory (HEI_TEG_, full set of reported household food items) were moderate to high, ranging from 0.42 (Sodium) to 0.83 (Total Fruit, Whole Fruit) (Table 3). Differences between the mean component and total scores were significant at *p* ≤ 0.003. The percent difference between the means ranged from −9% (Total Vegetables, Greens and Beans) to 17% (Solid Fats, Alcohol, Added Sugars) (Table 4). The highest practical differences in dietary equivalents were 0.15 cup/35 mL (Dairy) and 0.30 oz/8.50 g (Whole Grains).

### 3.5. Objective 4: Sensitivity and Range

**Figure 2:**
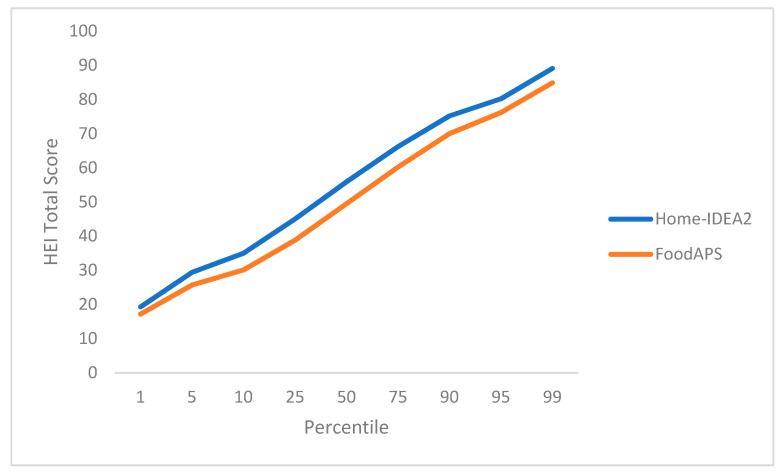
Percentile distribution of HEI total scores for the Home-IDEA2 and FoodAPS to test sensitivity and range between scores.

The component and total scores for the HEI_EEG_ representing the Home-IDEA2 limited inventory and the HEI_TEG_ representing the FoodAPS full inventory showed similar distributions across percentiles, demonstrating comparable range and sensitivity (Figure 2). Although the HEI_EEG_ total scores were consistently higher than the HEI_TEG_ total scores, the observed patterning was nearly identical. Some small variations were observed in the distribution of HEI component scores (Supplemental Table S1).

## 4. Discussion

In the present study, the capacity of the Home-IDEA2 to capture the quality of household food patterning was validated using multiple applications of the HEI-2010 algorithm using households from the FoodAPS dataset. For each measurement dimension tested, high correlations between the Home-IDEA2 HEI and FoodAPS HEI component and total scores demonstrated that: (1) EEG representing Home-IDEA2 items were appropriately calculated; (2) the food items selected to represent the Home-IDEA2 items sufficiently represented food items reported in the FoodAPS dataset; and (3) the HEI total score based on the subset of food items and EEG represented by the Home-IDEA2 was a reasonable proxy for the HEI total score based on actual food items and TEG reported in the FoodAPS dataset. The two instruments also showed reasonably comparable sensitivity and range for HEI total scores. The results from Objectives 1 and 2 establish that the comparisons made in Objectives 3 and 4 are reasonable to further test the utility of the Home-IDEA2 for measuring household food patterning and quality at the population level. Taken together, the findings from Objectives 3 and 4 demonstrate that the Home-IDEA2, a measurement tool that requires low participant and researcher burden, is a valid instrument for measuring the quality of household food availability, an important and modifiable aspect of the HFE that moves beyond the individual to an important environment in which individuals make many food choices.

With few exceptions, paired comparisons of the HEI component and total scores revealed significant differences between the scores applied to the Home-IDEA2 and FoodAPS inventories for the same households in each of the first three objectives. The significance of these differences was not altogether unexpected given the large sample size of the FoodAPS dataset and its ability to detect very small differences. Furthermore, when the practical differences (in dietary intake equivalents) between the Home-IDEA2 and FoodAPS inventories were compared, the differences translated to very small amounts, often less than 1/10th of a cup (<24 mL) or less than a one-ounce (<28 g) equivalent per 1000 calories.

Two component scores with the greatest differences between the Home-IDEA2 and FoodAPS inventories were Sodium and Solid Fats, Alcohols and Added Sugars (SoFAAS), where component scores representing the Home-IDEA2 limited inventory were consistently higher than scores representing the FoodAPS full inventories. It should be noted that the Home-IDEA2 was developed to capture maximal diversity of healthful foods in the household and is consequently weighted toward capturing raw and perishable foods that generally contribute to Adequacy components (whole fruit, total vegetables, greens and beans, whole grains, total dairy, total protein, and seafood and plant protein). Packaged and processed foods may be underrepresented in the Home-IDEA2, and these foods contribute to Moderation components and more specifically to Sodium and SoFAAS scores. Multiple comparisons of the Sodium and SoFAAS component scores revealed that the greatest differences were observed among food items rather than food amounts. This suggests that the quantities selected to represent reasonable purchase amounts were appropriate, and the items contributing to Sodium and SoFAAS scores were underrepresented by the Home-IDEA2. Indeed, many of the FoodAPS food items that did not map to the Home-IDEA2 included packaged foods such as canned soups, puddings, and ready-to-eat baked goods and sweets, which are typically high in added sodium and sugars. The 1482 food items reported in FoodAPS that did not map to the Home-IDEA2 are being reviewed and considered for inclusion in future versions of the Home-IDEA.

There are limitations to the present study. There were some differences in household characteristics between the present analytic sample and the full set of households included in the FoodAPS dataset. A lower proportion of households were from the South, had very low adult food security, had primary respondents who identified as Black, and had a high-school level of education. Among the FoodAPS limitations is the self-reported nature of the food-at-home component, which may have resulted in over- or underreporting of some types of foods. Additionally, there were some missing and inconsistent data in the FoodAPS dataset, such as households that reported no foods but were assigned food codes and reported foods with no corresponding food amounts. Because the intent of this project was to evaluate the ability of the Home-IDEA2 to represent an entire household food inventory, data in the FoodAPS dataset were used “as-is” for all households that had at least one food code with a corresponding food amount; no consideration was given to the sampling limitations within the FoodAPS data collection process. Therefore, the food items included present sample may not fully represent what is found in households across the U.S. An additional consideration is that the HEI-2010 is an older iteration of the HEI, yet it was selected for use in the present study because it represents the guidelines that were concurrent with the timing of data collection for FoodAPS. Therefore, as illustrated here, the scores representing the Home-IDEA2 inventories better capture adequacy versus moderation foods. This highlights an opportunity to improve the capture of moderation foods in future versions of the Home-IDEA.

Despite these limitations, there are strengths to this study in that data from the FoodAPS dataset represented U.S. households and actual food amounts. The FoodAPS dataset contained a sufficient number of households to examine how well the Home-IDEA2 represented household food patterns in the U.S., and using these data also provided an opportunity to examine the scope of food items missing from the Home-IDEA2. Finally, considerable effort was invested to model the database development and validation procedures using a process similar to that employed by the Center for Nutrition Policy and Promotion, U.S. Department of Agriculture in the development and validation of both the HEI-2005 and HEI-2010 [15,16].

## 5. Conclusions

Household food availability is an important, modifiable aspect of the HFE and therefore is a potential target for nutrition interventions that aim to improve individuals’ dietary quality and weight status. Yet food patterning is often burdensome to measure. In the present study, the ability of the low-burden Home-IDEA2 to accurately identify household food patterning and quality was validated against the FoodAPS dataset by applying the Healthy Eating Index-2010 (HEI-2010) in novel ways. These findings demonstrate that the Home-IDEA2 provides a valid measure of the HFE while reducing the burden of data collection for participants and researchers alike. Finally, because the Home-IDEA 2 enables measurement of a modifiable environment that exists above the level of the individual—one that is infrequently measured and closely tied to nutrition security—it has the potential to move researchers toward assessing population-based patterns of household-level access to food.

## Figures and Tables

**Table 1:** The healthy Eating Index 2010 component and total scores are density measures for dietary intake, as assessed by adherence to the 2010 Dietary Guidelines for Americans (DGA).

Concept ^a^	Component	Component Score Range	2010-DGA Recommended Intake Equivalent for Minimum and Maximum Scores	Dietary Equivalent of a 1-Point Score Increase ^b^
			Per 1000 Calories	
Adequacy	Total Vegetables ^c^	0–5	0 to ≥ 1.1 cup	+0.22 cup
Adequacy	Greens and Beans ^c^	0–5	0 to ≥ 0.2 cup	+0.04 cup
Adequacy	Total Fruit ^d^	0–5	0 to ≥ 0.8 cup	+0.16 cup
Adequacy	Whole Fruit ^e^	0–5	0 to ≥ 0.4 cup	+0.08 cup
Adequacy	Whole Grain	0–10	0 to ≥ 1.5 oz	+0.30 oz
Adequacy	Total Dairy ^f^	0–10	0 to ≥ 1.3 cup	+0.13 cup
Adequacy	Total Protein ^g^	0–5	0 to ≥ 2.5 oz	+0.50 oz
Adequacy	Seafood and Plant Proteins ^h^	0–5	0 to ≥ 0.8 oz	+0.16 oz
Adequacy	Fatty Acid Ratio ^i^	0–10	ratio ≤ 1.2 to ≥ 2.5	+0.13
Moderation	Sodium	0–10	≥ 2.0 to ≤ 1.1 g	−0.09 g
Moderation	Refined Grains	0–10	≥ 4.3 to ≤ 1.8 oz	−0.25 oz
Moderation	SoFAAS ^j^	0–20	≥50% to ≤19%	−1.55%
Adequacy and Moderation	Total Score ^k^	0–100		+1%

^a^ Adequacy is scored so that higher intakes result in higher scores, whereas moderation is reverse scored so that lower intakes result in higher scores. ^b^ A 1-point score increase represents an improvement in quality. Improvements in Adequacy are associated with increases in intake. Improvements in Moderation are associated with decreases in intake. ^c^ Includes any beans and peas not counted as Total Protein Foods. ^d^ Includes 100% fruit juice. ^e^ Includes all forms except juice. ^f^ Includes all milk products, including fluid milk, yogurt, cheese, and fortified soy beverages. ^g^ Beans/peas included in Total Protein (and not with vegetables) when Total Protein Foods standard is not met. ^h^ Includes seafood, nuts, seeds, soy products (no beverages), beans and peas counted as Total Protein Foods. ^i^ Fatty Acid Ratio uses the following formula: total unsaturated fats divided by total saturated fats [(total monounsaturated fat + total polyunsaturated fats)/total saturated fats]. ^j^ Calories from solid fats, alcohol, and added sugars; consumption cut point for including alcohol was >13 g/1000 kcal. ^k^ Sum of the component Scores.

**Table 2:** Household characteristics.

	Analytic Sample N = 4202 Households	Full Sample N = 4826 Households
	Mean ± SD N (%)	
Household Size ***	3.0 ± 1.7	2.9 ± 1.7
Family Size ***	2.8 ± 1.7	2.7 ± 1.7
Primary Respondent Hispanic	831 (20)	942 (20)
Primary Respondent Race ***		
White	3026 (72)	3371 (70)
Black	521 (12)	701 (15)
American Indian/Alaska Native	39 (1)	43 (1)
Asian/Native Hawaiian/Pacific Islander	191 (5)	215 (4)
Other Race	336 (8)	389 (8)
Multiple Races	84 (2)	101 (2)
Primary Respondent Education Level **		
10th grade or less	472 (11)	554 (11)
11th or 12th grade	241 (6)	284 (6)
High School Diploma/GED/Equivalent	1189 (28)	1388 (29)
Some college/Associate’s degree	1375 (33)	1575 (33)
Bachelor’s degree	624 (15)	697 (14)
Master’s degree or higher	298 (7)	325 (7)
Primary Respondent Employment		
Working at job/business	1875 (45)	2178 (45)
With job/business, not at work/on leave	114 (3)	129 (3)
Looking for work	345 (8)	407 (8)
Worked, looking for a job	35 (1)	38 (1)
Not working at job/business	1833 (44)	2074 (43)
Target Household Group		
Non-SNAP ^a^, <100% FPL ^b^	292 (7)	346 (7)
Non-SNAP, 100–185% FPL	745 (18)	851 (18)
Non-SNAP, ≥185% FPL	1807 (43)	2048 (42)
SNAP Recipient	1358 (32)	1581 (33)
WIC ^c^ Recipient in Household	411 (10)	461 (10)
Adult Food Security **		
High	2218 (53)	2522 (52)
Marginal	850 (20)	960 (20)
Low	665 (16)	785 (16)
Very Low	469 (11)	559 (12)
Household Food Sufficiency **		
Enough of foods wanted	2147 (51)	2452 (51)
Enough, not always the foods wanted	1661 (40)	1892 (39)
Sometimes not enough	302 (7)	365 (8)
Often not enough	92 (2)	117 (2)
Census Region **		
Northeast	711 (17)	816 (17)
Midwest	1019 (24)	1170 (24)
South	1523 (36)	1784 (37)
West	949 (23)	1056 (22)

** *p* < 0.01; *** *p* < 0.001 for significant differences between analytic and full FoodAPS samples. ^a^ SNAP: Supplemental Nutrition Assistance Program. ^b^ FPL: Federal Poverty Level. ^c^ WIC: Supplemental Nutrition Assistance Program for Women, Infants, and Children.

**Table 3:** Correlations between the Healthy Eating Index (HEI) component and total scores for each study objective ^a^.

	Objective 1 ^b^	Objective 2 ^c^	Objective 3 ^d^
	Examining Food Amounts (r)	Examining Food Types (r)	Examining Household Patterns (r)
Total Vegetables	0.76	0.87	0.74
Greens and Beans	0.81	0.81	0.60
Total Fruit	0.85	0.96	0.83
Whole Fruit	0.87	0.97	0.83
Whole Grains	0.93	0.92	0.63
Dairy	0.85	0.95	0.81
Total Protein	0.80	0.92	0.80
Seafood and Plant Protein	0.89	0.90	0.75
Fatty Acid Ratio	0.76	0.90	0.69
Sodium	0.64	0.62	0.42
Refined Grains	0.71	0.80	0.52
SoFAAS ^e^	0.74	0.81	0.57
Total Score	0.75	0.88	0.71

^a^ Pearson’s correlations. All except Objective 1 Sodium (*p* = 0.01) and Fatty Acid Ratio (*p* = 0.77) are significant at *p* < 0.003. ^b^ Objective 1 compared estimated edible grams (HEI_EEG_) to reported total edible grams (HEI_TEG_) for the subset of 104 representative items in the Home-IDEA2 inventory (N = 4074 households). ^c^ Objective 2 compared all foods that mapped to the subset of food items represented by the Home-IDEA2 inventory to the full inventory (including those that did not map to the Home-IDEA2) reported in FoodAPS. Both sets of HEI scores were calculated based on TEG (HEI_TEG_) (N = 4202 households). ^d^ Objective 3 compared the estimated edible grams for the subset of food items represented by Home-IDEA2 inventory (HEI_EEG_) to the total edible grams for the full household inventory reported in FoodAPS (HEI_TEG_) (N = 4202 households). ^e^ SoFAAS: Solid Fats, Alcohol, Added Sugars.

**Table 4:** Means, percent differences, and dietary intake equivalents for the Healthy Eating Index (HEI) component and total scores for each study objective.

Component	Instrument	Objective 1 ^a^			Objective 2 ^b^			Objective 3 ^c^		
		Examining Food Amounts			Examining a Food Types			Examining the Household Patterns		
		Score Mean (SD)	Percent Difference ^d^	Dietary Intake Equivalent ^e^	Score Mean (SD)	Percent Difference ^d^	Dietary Intake Equivalent ^e^	Score Mean (SD)	Percent Difference ^d^	Dietary Intake Equivalent ^e^
Total Vegetables	Home-IDEA2 ^g^	2.2 (2.1) *	7%	0.07 cup	2.4 (2.0) *	−4%	0.05 cup	2.2 (2.0) *	−9%	0.10 cup
	FoodAPS ^h^	1.9 (2.2)			2.6 (1.9)			2.6 (1.9)		
Greens and Beans	Home-IDEA2	0.7 (1.7) *	3%	0.01 cup	1.2 (2.0) *	−5%	0.01 cup	0.9 (1.9) *	−9%	0.02 cup
	FoodAPS	0.5 (1.5)			1.4 (2.1)			1.4 (2.1)		
Total Fruit	Home-IDEA2	2.6 (2.3) *	5%	0.04 cup	2.4 (2.1) *	5%	0.04 cup	2.6 (2.1) *	9%	0.08 cup
	FoodAPS	2.3 (2.3)			2.2 (2.0)			2.2 (2.0)		
Whole Fruit	Home-IDEA2	2.8 (2.4) *	6%	0.02 cup	2.4 (2.2) *	3%	0.01 cup	2.7 (2.3) *	9%	0.03 cup
	FoodAPS	2.5 (2.4)			2.3 (2.1)			2.3 (2.1)		
Whole Grains	Home-IDEA2	1.3 (3.2) ^i^*	0%	0.01 oz (0.28 g)	2.4 (3.6) *	0%	0.01 oz (0.34 g)	3.4 (4.1) *	10%	0.30 oz (8.50 g)
	FoodAPS	1.3 (3.2)			2.4 (3.3)			2.4 (3.3)		
Dairy	Home-IDEA2	5.8 (4.6) *	7%	0.09 cup	5.6 (4.1) *	5%	0.07 cup	6.2 (4.0) *	12%	0.15 cup
	FoodAPS	5.1 (4.7)			5.1 (3.8)			5.1 (3.8)		
Total Protein	Home-IDEA2	1.9 (2.1) *	4%	0.10 oz (2.94 g)	2.8 (2.1) ^l^*	1%	0.03 oz (0.98 g)	3.1 (2.1) *	6%	0.16 oz (4.48 g)
	FoodAPS	1.7 (2.1)			2.8 (2.0)			2.8 (2.0)		
Seafood and Plant Protein	Home-IDEA2	0.8 (1.8) ^j^*	1%	0.01 oz (0.28 g)	1.7 (2.2) *	4%	0.03 oz (0.84 g)	1.5 (2.1) *	−8%	0.06 oz (1.70 g)
	FoodAPS	0.8 (1.8)			1.9 (2.2)			1.9 (2.2)		
Fatty Acid Ratio	Home-IDEA2	4.4 (4.4) ^k^	1%	0.02	4.7 (4.2) *	−1%	0.02	4.4 (4.1) *	−4%	0.06
	FoodAPS	4.3 (4.4)			4.9 (4.1)			4.9 (4.1)		
Sodium	Home-IDEA2	8.1 (3.3)	0%	0.00 g	7.8 (3.2) *	11%	0.10 g	8.0 (2.9) *	12%	0.11 g
	FoodAPS	8.1 (3.3)			6.7 (3.8)			6.7 (3.8)		
Refined Grains	Home-IDEA2	5.8 (4.6) *	−6%	0.15 oz (4.25 g)	7.2 (3.8) *	5%	0.13 oz (3.68 g)	6.0 (4.1) *	6%	0.15 oz (4.25 g)
	FoodAPS	6.4 (4.4)			6.6 (3.8)			6.6 (3.8)		
SoFAAS ^f^	Home-IDEA2	15.2 (6.9) *	2%	0.62% energy	12.7 (7.2) *	9%	2.84% energy	14.3 (6.5) *	17%	5.21% energy
	FoodAPS	14.8 (7.4)			10.9 (7.3)			10.9 (7.3)		
Total Score	Home-IDEA2	51.6 (16.1) *	2%	2.0 points	53.5 (15.8) *	4%	3.7 points	55.5 (15.7) *	6%	5.7 points
	FoodAPS	49.6 (18.1)			49.8 (15.4)			49.8 (15.4)		

^a^ Objective 1 compared estimated edible grams (HEI_EEG_) to reported total edible grams (HEI_TEG_) for the subset of 104 representative items in the Home-IDEA2 inventory (N = 4074 households). ^b^ Objective 2 compared all foods that mapped to the subset of food items represented by the Home-IDEA2 inventory to the full inventory (including those that did not map to the Home-IDEA2) reported in FoodAPS. Both sets of HEI scores were calculated based on TEG (HEI_TEG_) (N = 4202 households). ^c^ Objective 3 compared the estimated edible grams for the subset of food items represented by Home-IDEA2 inventory (HEI_EEG_) to the total edible grams for the full household inventory reported in FoodAPS (HEI_TEG_) (n = 4202 households). ^d^ Percent Difference calculations use FoodAPS as the referent. Positive values indicate the Home-IDEA2 score is higher than the FoodAPS score, whereas negative values indicate the Home-IDEA2 score is lower than the FoodAPS score. ^e^ Dietary Intake Equivalent is the relative measure of the intake amount associated with the percent difference between means. The referent is the dietary intake requirement per 1000 calories to receive a maximum score for a given component. ^f^ SoFAAS: Solid Fats, Alcohol, Added Sugars. ^g^ Home-IDEA2: Home Inventory for Describing Eating and Activity, Version 2. ^h^ FoodAPS: National Food Acquisition and Purchase Survey. ^i^ Whole Grains Objective 1 Mean (SD) for Home-IDEA2 1.31 (3.18); FoodAPS 1.26 (3.17); t = −2.725, df = 4073. ^j^ Seafood and Plant Protein Objective 1 Mean (SD) for Home-IDEA2 0.85 (1.81); FoodAPS 0.78 (1.78); t = −5.353, df = 4073. ^k^ Fatty Acid Ratio Objective 1 Mean (SD) for Home-IDEA2 4.43 (4.42); FoodAPS 4.31 (4.45); t = −2.472, df = 4073. ^l^ Total Protein Objective 2 Mean (SD) for Home-IDEA2 2.85 (2.10); FoodAPS 2.78 (2.02); t = −5.580, df = 4201. * The difference between the means is significant at *p* < 0.003.

## Data Availability

Data are available upon request from the corresponding author.

## Supplementary Materials

The following are available online at https://www.mdpi.com/article/10.3390/nu13113930/s1, Table S1. Estimated Home-IDEA2^a^ and FoodAPS^b^ HEI Component and Total Score Means at each Percentile.
