# Supplementary material for: Validation of a Home Food Environment Instrument Assessing Household Food Patterning and Quality
Source: Nutrients. 2021 Nov 3;13(11):3930. doi: 10.3390/nu13113930 (PMC8619541; doi:10.3390/nu13113930)
Supplement: Supplementary file 1 [file nutrients-13-03930-s001.zip › nutrients-1418825-supplementary.pdf]

Table S1. Estimated Home-IDEA2[a] and FoodAPS[b] HEI Component and Total Score Means at each Percentile

Components	Quality Score / Tool	Mean (SD)	Percentiles[c]								
			1st	5th	10th	25th	50th	75th	90th	95th	99th
Total Vegetables	Home-IDEA2	2.2 (2.0)	0.0	0.0	0.0	**0.0**	1.7	**4.4**	5.0	5.0	5.0
	FoodAPS	2.6 (1.9)	0.0	0.0	0.0	**0.7**	2.4	**5.0**	5.0	5.0	5.0
Greens & Beans/Peas	Home-IDEA2	0.9 (1.9)	0.0	0.0	0.0	0.0	0.0	**0.0**	5.0	5.0	5.0
	FoodAPS	1.4 (2.1)	0.0	0.0	0.0	0.0	0.0	**3.3**	5.0	5.0	5.0
Total Fruit	Home-IDEA2	2.6 (2.1)	0.0	0.0	0.0	**0.0**	2.8	**5.0**	5.0	5.0	5.0
	FoodAPS	2.2 (2.0)	0.0	0.0	0.0	**0.1**	1.7	**4.5**	5.0	5.0	5.0
Whole Fruit	Home-IDEA2	2.7 (2.3)	0.0	0.0	0.0	0.0	3.6	5.0	5.0	5.0	5.0
	FoodAPS	2.3 (2.1)	0.0	0.0	0.0	0.0	1.8	5.0	5.0	5.0	5.0
Whole Grain	Home-IDEA2	3.4 (4.1)	0.0	0.0	0.0	0.0	**0.0**	7.5	**10.0**	10.0	10.0
	FoodAPS	2.4 (3.4)	0.0	0.0	0.0	0.0	**0.3**	4.0	**9.1**	10.0	10.0
Total Dairy	Home-IDEA2	6.2 (4.0)	0.0	0.0	0.0	2.5	7.6	**10.0**	10.0	10.0	10.0
	FoodAPS	5.1 (3.8)	0.0	0.0	0.0	1.2	4.9	**9.5**	10.0	10.0	10.0
Total Protein	Home-IDEA2	3.1 (2.1)	0.0	0.0	0.0	0.4	4.1	5.0	5.0	5.0	5.0
	FoodAPS	2.8 (2.0)	0.0	0.0	0.0	0.7	3.0	5.0	5.0	5.0	5.0
Seafood & Plant Proteins	Home-IDEA2	1.5 (2.1)	0.0	0.0	0.0	0.0	**0.0**	**4.0**	5.0	5.0	5.0
	FoodAPS	1.9 (2.2)	0.0	0.0	0.0	0.0	**0.5**	**5.0**	5.0	5.0	5.0
Fatty Acid Ratio	Home-IDEA2	4.4 (4.1)	0.0	0.0	0.0	**0.0**	3.6	**9.8**	10.0	10.0	10.0
	FoodAPS	4.9 (4.1)	0.0	0.0	0.0	**0.2**	4.6	**10.0**	10.0	10.0	10.0
Sodium	Home-IDEA2	8.0 (2.9)	0.0	**0.8**	3.1	6.7	**10.0**	10.0	10.0	10.0	10.0
	FoodAPS	6.7 (0.1)	0.0	**0.0**	0.0	3.7	**8.6**	10.0	10.0	10.0	10.0
Refined Grains	Home-IDEA2	6.0 (4.1)	0.0	0.0	0.0	1.7	7.4	10.0	10.0	10.0	10.0
	FoodAPS	6.6 (3.9)	0.0	0.0	0.0	3.4	8.6	10.0	10.0	10.0	10.0
SoFAAS[d]	Home-IDEA2	14.3 (6.5)	0.0	0.0	**2.8**	10.5	16.5	**20.0**	20.0	20.0	20.0
	FoodAPS	10.9 (7.3)	0.0	0.0	**0.0**	4.3	11.6	**18.2**	20.0	20.0	20.0
Total Score	Home-IDEA2	55.5 (15.7)	19.3	29.4	35.0	45.0	55.9	66.2	75.2	80.2	89.1
	FoodAPS	49.8 (15.4)	17.2	25.7	30.1	38.8	49.5	60.2	70.0	76.2	84.9

[a] Home-IDEA2: Home Inventory Describing Eating and Activity, Version 2. HEI score calculated based on representative food codes and estimated edible grams.

[b] FoodAPS: National Food Acquisition and Purchase Survey. HEI score calculated based on full inventory of reported food codes and total edible grams.

[c] Bold numbers indicate that the lower or upper range appear at different percentiles.

[d] SoFAAS: Solid Fats, Alcohol, Added Sugars
